# Development of Mineral-Bonded Plywood with Magnesium Oxychloride as a Binder Using the Hot-Pressing Process

**DOI:** 10.3390/polym15040805

**Published:** 2023-02-05

**Authors:** Ali Shalbafan, Heiko Thoemen

**Affiliations:** 1 Department of Wood and Paper Science and Technology, Faculty of Natural Resources and Marine Sciences, Tarbiat Modares University, Noor P.O. Box 46414-356, Iran; 2 Institute of Materials and Wood Technology, School of Architecture, Wood and Civil Engineering, Bern University of Applied Sciences, 2504 Biel/Bienne, Switzerland

**Keywords:** plywood, mineral binder, magnesium oxide, petroleum-free binder, hot-press, no-added formaldehyde

## Abstract

Environmentally friendly plywood panels were produced by a hot-pressing process using magnesium oxychloride cement (MOC) as a no-added formaldehyde adhesive. Magnesium oxychloride cement binders were prepared with different molar ratios of MgO:MgCl_2_ (M/C) and H_2_O:MgCl_2_ (W/C) ranging from 6 to 12 and 15 to 21, respectively, for plywood production. The binder properties measured were gel time, differential scanning calorimetry (DSC) and Fourier transom infrared spectroscopy (FTIR). The quality of the plywood panels was analyzed based on their mechanical (shear and bending) and physical (thickness swelling and water absorption) properties. A positive effect on the properties of the MOC binder as well as on the properties of the plywood was observed by increasing the molar ratio M/C up to a value of 9. The shear and flexural properties of the plywood specimens were negatively affected by further increasing the molar ratio M/C to 12 and the molar ratio W/C from 15 to 21. Differential scanning calorimetry analysis showed a peak temperature of less than 100 °C for MOC curing, which meets the requirements of hot press technology. No delamination of the plywood specimens was observed after 24 h immersion in tap water or 6 h immersion in boiling water and after a cyclic delamination test. In general, mineral-bonded plywood with magnesium oxychloride shows promising properties for indoor and outdoor use, although the binder quality should still be improved.

## 1. Introduction

The development of petroleum-free binders has attracted the interest of researchers around the world, as it is seen as an important step towards the sustainable development of the wood industry [1,2]. Fossil-based adhesives, especially those based on formaldehyde, dominate the bonding of engineered wood products (EWP) due to their high reactivity, fast curing, low cost and sufficient properties [3]. However, the long-term decreasing availability of fossil resources on the one hand and the increasing environmental concerns related to climate change on the other hand promote the development of petroleum-free binders for EWP manufacturing. In addition, new and more restrictive regulations on the emission of volatile organic compounds, in particular formaldehyde as a carcinogenic substance, are encouraging the development of EWP with no added formaldehyde adhesive [3,4]. In this context, mineral (inorganic) binders can address the above challenges with a novel and radical approach. Mineral binders can be considered as a multifunctional binder system as they do not release formaldehyde from the binder and have high fire, mold and fungus resistance, which makes them more attractive compared to fossil-based adhesives and other bio-based alternatives [5,6,7].

Gypsum and Portland cement are traditional mineral constituents used extensively in the manufacture of wood–mineral composites [8,9,10]. Although these composites have a similar shape to conventional EWP, they have a wood content of less than 50% and a panel density of about 1100 kg/m^3^, which is far from the level required for conventional EWP. In recent years, some research groups have been working on the substitution of organic binders by mineral binders for the reasons explained above. For example, Portland cement has been used as a binder in the manufacture of plywood panels [11]. Berzins et al. [12] have shown that geopolymers can be used for the gluing of solid wood. Researchers also successfully demonstrated that the organic binder can be replaced by a mineral binder for the laboratory production of plywood and LVL [13,14,15]. In these works, geopolymer was formulated as a mineral binder using aluminosilicate powder and an alkaline activator prepared from a mixture of sodium silicate and potassium hydroxide. However, the use of these mineral binders in conventional EWP is associated with some technological problems in panel production, such as their high alkalinity and the silicon content of the binder [6,16]. To overcome these problems, Sorel or magnesium oxychloride cement (MOC) has recently gained more interest [17].

The Sorel or MOC binder, which consists of magnesium oxide (MgO) in powder form (called magnesia) and magnesium chloride (MgCl_2_), was first described by the French scientist Stanislas Sorel in 1866 [7]. Compared to previously used mineral binders, MOC as a binder is preferable due to its lower pH, lower sensitivity to wood pH, lower viscosity, lighter color and silica-free ingredients [18,19]. A composite product with a density of 1000 kg/m^3^ was prepared from a mixture of wood and MOC using an extrusion process [20]. The MOC was also used as an inorganic adhesive for the production of flame-retardant plywood by the cold-pressing method [17,21]. It has been stated that the ratio of components in the MOC binder plays an important role on the characteristics of the final hydrated product [22]. The effects of molar ratios (MgO/MgCl_2_ and H_2_O/MgCl_2_) and curing conditions on MOC were investigated [23]. The results show that the use of appropriate molar ratios and curing conditions can significantly improve the MOC features [24]. Magnesium oxychloride cement products are considered to have excellent properties when the molar ratio of MgO/MgCl_2_ is varied between 5 and 10 [18,19]. In one study, the best mechanical properties of MOC products were obtained at a molar ratio of about 7 [17]. The curing of MOC at different temperatures between 0 °C and 65 °C was also investigated. It was shown that the curing at temperatures between 20 °C and 50 °C resulted in the most stable MOC under humid conditions [25]. When curing occurs at a higher temperature, a certain amount of unreacted MgO remains in the system [24].

The researchers also used polyacrylamide and sodium polyacrylate in a blend with the MOC binder to overcome the low initial viscosity and poor water resistance of the MOC binder for plywood production [21]. However, most of the developed mineral binders and the corresponding EWP are still far from massive industrial implementation due to their cold-pressing technology. In the last 80 years, hot-pressing technology has dominated the field of EWP production because it allows massive and more economical production [26]. Actually, hot-pressing technology is the most critical step of the whole board manufacturing, as the properties of the final product strongly depend on the pressing conditions of the mat [26]. Initial approaches to the application of mineral binders (i.e., geopolymer) in EWP using hot-pressing technology similar to that of conventional EWP have been presented by researchers [13,15]. They found that the mineral binder, which is hardened in the hot press, works differently than that of cold pressing.

All in all, on the one hand, mineral binders for solid wood or veneer panels are being developed, and on the other hand, mineral fillers are more accepted by the EWP industry as wood substitutes [27]. It is therefore likely that the development of mineral binders will also be soon addressed by the industry. Among the various types of mineral binders studied, MOCs have great potential for EWP production, but their behavior at higher curing temperatures has not been investigated. Moreover, the effects of the molar ratio of the components used to produce MOC using hot press technology to harden have rarely been investigated, which is the focus of the current study. In this study, MOC binders with different molar ratios of MgO:MgCl_2_ (M/C from 6 to 12) and H_2_O:MgCl_2_ (W/C from 15 to 21) were prepared and their effects on the properties of hot-pressed plywood were observed.

## 2. Materials and Methods

### 2.1. Materials

Peeled poplar (Populus) veneers without defects and with uniform surface were purchased from Hess & Co., AG (Döttingen, Switzerland). Veneers with average density, thickness and moisture content of 451 kg/m^3^, 3.1 mm and 7.8%, respectively, were used for the plywood manufacturing.

Finely ground natural magnesium oxide (MgO, Magnesia 312) with a particle size of less than 120 mesh was purchased from Magnesia GmbH (Lüneburg, Germany). The MgO was caustic burnt with white to light brown color. Technical magnesium chloride-6-hydrate, abbreviated as magnesium chloride in this study (MgCl_2_, Magnesia 4151), was also purchased from Magnesia GmbH (Lüneburg, Germany). Deionized water was also used for the production of the MOC binder. The chemical composition of the MgO and MgCl_2_·6H_2_O is shown in Table 1 and Table 2, respectively.

### 2.2. Production of MOC Binder

To prepare the MOC binder, an appropriate amount of deionized water and magnesium chloride was first whisked at room temperature for 10 min at about 1000 rpm. The MgO powder was then gradually added to the solution and whisked at about 1000 rpm for another 10 min until a homogeneous mixture was obtained. In this study, MOC binders with different molar ratios of binder components (M/C and W/C) were prepared for the production of plywood, as shown in Table 3. The preliminary tests showed that it is difficult to distribute the binder on the veneer surfaces with lower and higher solids content than tested in this study, so the test setup was adjusted as shown in Table 3.

The molar ratio of MgO:MgCl_2_ (M/C) was varied between 6 and 12, while the molar ratio of H_2_O:MgCl_2_ (W/C) was kept constant at 18 (including the bound water in magnesium chloride-6-hydrate). Consequently, the ratio of W/C was changed from 15 to 21, while the molar ratio of M/C was kept constant at 9. For simplicity, binders with different molar ratios of MgO:MgCl_2_ and H_2_O:MgCl_2_ were denoted as M/C and W/C, respectively. The final solids content of the MOC binder varies between about 51% and 64% depending on the molar ratio of the binder components.

### 2.3. Production of Plywood

**Figure 1:**
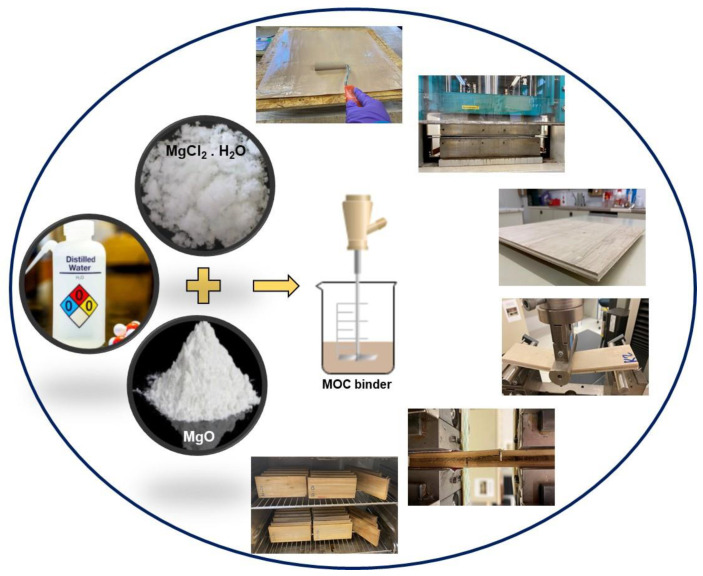
Manufacturing process for MOC binder and the plywood panels made from MOC binder.

Three-ply plywood sheets with dimensions 500 × 500 × 9 mm^3^ were produced. The MOC binder was applied to the surface of the veneers with a roller, then the veneers were aligned layer by layer perpendicular to the plywood panels. The veneer mat was then pressed in a single opening laboratory hot press (Höfer HLOP 210, Höfer Presstechnik GmbH, Taiskirchen, Austria). A pressing temperature of 140 °C, a pressing pressure of 1.5 MPa and a pressing factor of 55 s/mm were used for hot pressing, resulting in a pressing time of 495 s for all panels. These parameters were chosen in view of our previous experience with mineral binders as well as the preliminary tests [14,15]. A similar hot-pressing schedule was used for the production of all panels. The manufacturing process for MOC-bonded plywood panels is shown in Figure 1.

The binder was applied to the veneer surfaces with a roller, as shown in Figure 1. The application rate of the dry MOC binder was kept constant at 420 g/m^2^ for all plywood panels, while the solid content of the binder varied in the different formulations. The mineral binder has higher density compared to conventional adhesive; hence, a higher amount of binder is needed to meet the full coverage of veneer faces [14]. The average density of the plywood panels made with MOC binder was about 560 kg/m^3^. Three plywood panels were made for each of the variables, resulting in a total of twenty-seven panels. The manufactured plywood panels were conditioned at a relative humidity of 65 ± 3% and a temperature of 20 ± 2 °C for two weeks prior to testing.

### 2.4. Binder Characterization

To evaluate the influence of the molar ratio of M/C and W/C on the reactivity of the MOC binder, the gel time of selected MOC binders was measured at a temperature of 100 °C using a gel time meter from Gelnorm instrumente AG (Oberuzwil, Switzerland). The gel time of the binder was determined as the elapsed time during which the binder could not be further stirred. Before measuring the gel time, the pH of the MOC binder was measured with different M/C and W/C molar ratios at room temperature using a Mettler Toledo digital pH meter (SevenMulti, Greifensee, Switzerland). The gel time and pH measurement were repeated three times for each MOC binder formulation.

Differential scanning calorimetry (DSC) was performed to evaluate the curing behaviour of the MOC binder with different binder formulations. The DSC was performed using the DSC6000 from Perkin Elmer (Waltham, MA, USA) with high pressure cells. For DSC measurement, about 10 mg of the solid MOC binder was added to the cells. Measurements were made in the range of 25 °C and 200 °C at a heating rate of 5 °C. The parameters analyzed after DSC measurement were initial temperature, peak temperature and heat of reaction. By integrating the exothermic peak area, the heat of reaction, expressed in joules per gram (J/g) of solids content of the binder, was calculated. A urea-formaldehyde resin (UF) from BASF (Kaurit Glue 345, Ludwigshafen, Germany) with a solids content of 67% was used as a reference and for comparison with MOC binder properties.

The effects of the different molar ratios of the components on the MOC binder were analyzed by Fourier transform infrared spectroscopy (FTIR) in attenuated total internal reflection mode. The MOC binder cured at a temperature of 100 °C was completely ground. The spectrogram of the MOC samples was generated using a Perkin Elmer spectrometer (Waltham, MA, USA). The spectra were recorded with a resolution of 4 cm^−1^ in the range of 4000–550 cm^−1^.

### 2.5. Plywood Characterization

The service class of plywood depends on the quality of bonding, which is determined according to EN 314-1. In this study, the suitability of the specimens for Class 1 (indoor conditions) and Class 2 (covered outdoor conditions) was evaluated. Therefore, the plywood specimens were subjected to treatments A and B (according to EN314-1) described as follows before shear testing:

(A) Immersion for 24 h in tap water (at 20 ± 3 °C);
(B) Immersion for 6 h in boiling water followed by cooling in tap water (at 20 ± 3 °C) for at least 1 h.

The untreated samples (so-called dry samples) were also tested for comparison purposes to evaluate the effects of the above treatments on the bonding quality of MOC-bonded plywood panels. The treated and untreated specimens were tested on a Zwick universal testing machine (Zwick Roell Group, Ulm, Germany) at a constant crosshead displacement of 0.5 mm/min in shear mode. The reported mean value of shear strength corresponds to the average of 12 specimens for each binder formulation.

The flexural properties of the panels were evaluated by measuring the flexural strength (MOR) and modulus of elasticity (MOE) according to EN 310 on specimens aligned parallel to the fibers of the face sheets. The dimensions of the flexural specimens were 230 × 50 × 9 mm^3^ (length × width × thickness). The tests were performed at a constant crosshead displacement rate of 8 mm/min on a Zwick universal testing machine.

Delamination testing was performed using the three-cycle soak test described in ANSI/HPVA HP-1 (2020). For this purpose, three specimens (50.8 mm × 127 × 9 mm^3^) from each panel variable were exposed to three consecutive cycles in tap water at 24 ± 3 °C for 4 h and then dried in an oven at 50 ± 2 °C with sufficient air circulation. The specimen is acceptable for indoor and outdoor use, respectively, if <5% and >15% of the specimens are delaminated after the first and third soak/dry cycles. Delamination is defined in ANSI/HPVA HP-1 (2020) as any continuous opening between two layers of plywood that is longer than 5.08 cm, deeper than 0.64 cm and wider than 0.008 cm.

Thickness swelling (TS) was determined after 2 and 24 h of water immersion according to EN 317 (specimen size 50 × 50 mm^2^). The water absorption (WA) of the specimens was also calculated based on the absorbed water content after 2 and 24 h of water immersion. The mean value of TS and WA given is the average of 10 samples for each binder formulation. A one-way ANOVA test was performed using SPSS software (IBM SPSS Statistics 25) to evaluate the changes in physical and mechanical properties between the different binder formulations. Statistical significance was set at *p* < 0.05.

## 3. Results and Discussion

### 3.1. Binder Characterization

Varying the molar ratio of M/C and W/C showed no significant change in the pH of MOC binders (Table 4). The pH of the MOC binders was about 8.5 in all binder formulations, which is close to the level of conventional UF resin. The pH value of the binder plays an important role in the panel characteristics. The moisture-related behaviour of EWP can be influenced by the types of adhesives, which have different sorption behaviour. For example, alkali-containing resins such as phenol formaldehyde are highly hygroscopic and absorb more water than UF-bonded boards under similar conditions. In addition, the higher alkalinity of the binder can lead to increasing damage to the cell structures of the wood (i.e., damage to lignin and hemicelluloses), which in turn creates weak points for delamination [5,28]. Therefore, a moderate pH in MOC binders, similar to conventional UF, would be beneficial for their processability and industrial use.

Table 4 shows the setting time (gel time) of the MOC binders with different M/C and W/C molar ratios of the components. By doubling the molar ratio of M/C from 6 to 12, the setting time of the binder was shortened by about 25% and reduced from 155 s to 116 s. By increasing the molar ratio of W/C from 15 to 21, the setting time was extended by about 15% from 124 s to 142 s. The reason for these changes could be related to the change in the solids content of the binder. The increase in the molar ratios of M/C and W/C increased and decreased the solids content of the binder, respectively. The results showed that the setting time of the MOC binder was shortened by increasing the solid content of the binder. The gel time of an organic adhesive such as UF resin is also influenced by the binder solids content 13]. For organic adhesives, the gel time decreases with increasing solids content, which is due to the increase in resin concentration with increasing solids content. Therefore, with more water in the system, the binder is more diluted and affects the curing reactions and acts as an energy barrier for the curing of the resin. This reduces the curing speed, resulting in a longer gel time [29]. Table 4 also shows that the MOC binder has a much faster setting and reaction time compared to the UF binder. We must take into account that UF and MOC are completely different binder systems and have different reaction kinetics. Therefore, this comparison may not be definitive and further investigation may be required.

Table 4 also presents the data obtained from the DSC analysis. The onset temperature was not significantly changed by different molar ratios of M/C and W/C in the MOC binder formulation. The peak temperature was also not affected by the change in M/C and W/C molar ratio and was almost the same for all binder formulations at about 98 °C. The initial and peak temperatures of the UF binder as a reference were measured to be 77.5 °C and 92.8 °C, respectively, which are similar to the temperatures of the MOC binders. This is an important finding, showing that the determination of hot press parameters to produce MOC-bonded plywood is feasible, such as UF-bonded panels.

**Figure 2:**
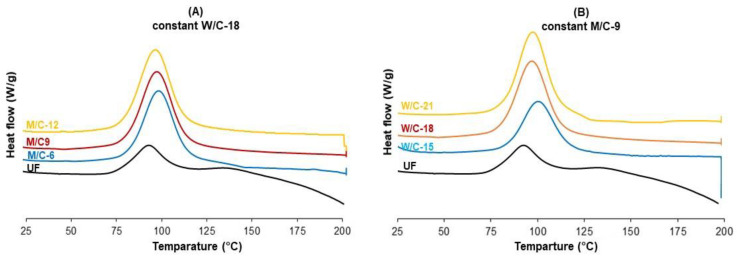
**The** DSC curves of the MOC binders with different molar ratios, (**A**) different MgO:MgCl_2_ ratio, (**B**) different H_2_O:MgCl_2_ ratio.

**Figure 3:**
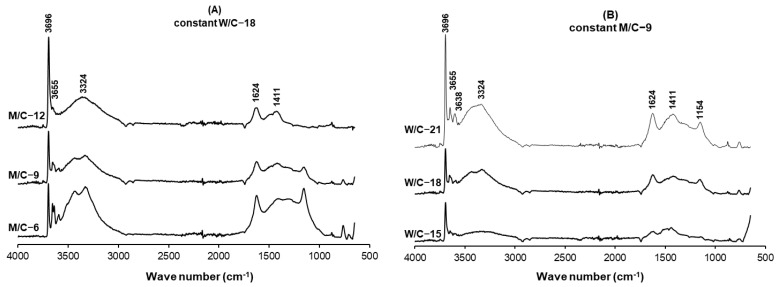
The ATR-FTIR curves of the MOC binders with different molar ratios, (**A**) different MgO:MgCl_2_ ratio, (**B**) different H_2_O:MgCl_2_ ratio.

Magnesium oxychloride cement binders exhibit an exothermic reaction that contributes most to heat generation in DSC measurements (Figure 2). The addition of MgO to the binder formulation (increasing the M/C molar ratio) slightly increases the thermal heat of reaction from 342 J/g to about 375 J/g. However, increasing the W/C molar ratio from 15 to 21 increased the heat of the reaction by about 70%, rising from about 270 J/g to 461 J/g. The influence of excess water on the heat of the reaction of MOC curing could be explained by the formation of a higher magnesium hydroxide content in the system, as can also be seen later in Figure 3. Magnesium hydroxide (Mg(OH)_2_) is formed by the combination reaction of magnesium oxide (MgO) with ionized water, which typically takes place at a temperature well above room temperature, i.e., about 120 °C, which is possible with the hot-pressing technique [24]. In general, it can be said that the molar ratio of the binder components significantly affects the thermal reaction heat of the MOC binder reaction. 

The structural changes of the different MOC binder formulations were investigated using the FTIR technique (Figure 3). The peaks at 3696 cm^−1^, 3655 cm^−1^, 3638 cm^−1^ and 3592 cm^−1^ originated from the Mg-OH bending vibrations of the phase-5 crystal [21]. Some of these peaks disappeared or their intensity decreased when the M/C molar ratio was increased from 6 to 12 and the W/C molar ratio was decreased from 21 to 15. Excess MgO and H_2_O in the binder could lead to the formation of Mg(OH)_2_, resulting in instability and a possible reduction in binder strength [23]. The bands at 3300 to 3500 cm^−1^ and at 1624 cm^−1^ can be attributed to the O-H bending vibrations and H-O-H bending moment of the crystal water, respectively [30]. The intensity of these peaks decreased with increasing the molar ratio of M/C and decreasing W/C, which is attributed to the reduced water in the system when these molar ratios are changed. The tiny peaks in the 1800–3000 cm^−1^ range caused by the symmetric and asymmetric stretching modes of the O-H bonds in H_2_O and Mg(OH)_2_ can be attributed to the further thermal stress to the cured binder in the hot press [31]. Moreover, the absorption bands at 1154 cm^−1^ are due to the weak bonding vibration of Cl-O, which disappeared in the samples with M/C-12 and W/C15 [17,31]. The further addition of light burnt MgO to the binder formulation (increasing the M/C molar ratio to 12) could cause the excess MgO to react more with the water molecules to form more Mg(OH)_2_, as shown by the sharper peak at 3696 cm^-1^ associated with Mg(OH)_2_ in the M/C-12 sample. On the other hand, the lower water content in the W/C-15 formulation could be another reason for the disappearance of the Cl-O bond in the cured binder. The peak at about 850 cm^−1^ is attributed to the stretching vibration of Mg-O in a cubic structure.

### 3.2. Mechanical Properties

#### 3.2.1. Shear Strength Analysis

**Figure 4:**
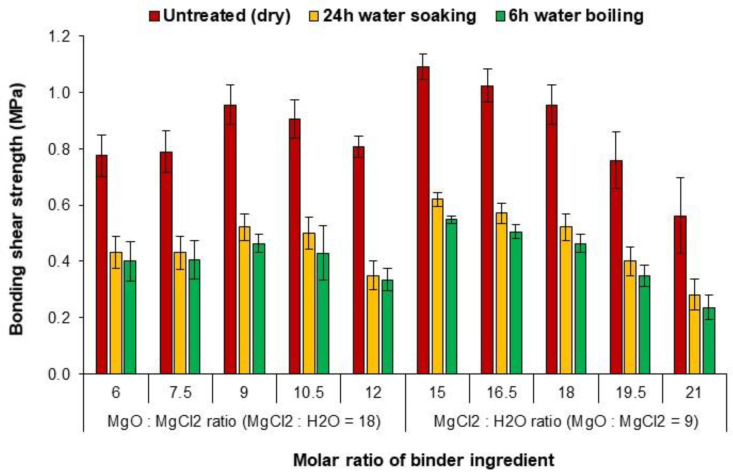
Shear strength of plywood samples using MOC binders with different molar ratios of the components.

The bond shear strength of MOC binders with various molar ratios of M/C and W/C is presented in Figure 4. As shown, the shear strength is significantly increased by increasing the magnesium oxide content in the binder formulation (M/C molar ratio) up to an M/C ratio of 9. This could be due to the denser binder microstructure resulting from the increase in the M/C molar ratio. In addition, the unreacted MgO can serve to some extent as a filler in the MOC structure to fill the voids and improve the binder strength [17]. However, further increasing the M/C ratio up to a value of 12 had a negative impact on the bonding shear strength, resulting in a 15% and 30% decrease in shear strength for the untreated and treated specimens, respectively. As shown by the FTIR analysis, the excess of MgO in the binder led to the formation of more Mg(OH)_2_, which may lead to an increase in the stress of the binder, instability and a decrease in the binder strength [18].

Increasing the W/C molar ratio from 15 to 21 while maintaining the M/C ratio of 9 results in a significant decrease in the shear strength of the binder. In other words, the lower the water content, the higher the bonding shear strength of the MOC binder. This was because the excess H_2_O reacted more with MgO, resulting in a higher content of Mg(OH)_2_, which reduced the strength of the binder [25]. Excess water could also have caused pores and contributed to the observed reduction in strength [23]. It is also conceivable that the binder with lower solids content (higher W/C molar ratio) penetrated more into the wood cells/cavity structure and resulted in a weak glue joint (starved glue joint) [14,15,29]. The highest bond shear strength was observed for samples with M/C and W/C molar ratios of 9 and 15, respectively, with 1.1 MPa for the untreated sample, 0.62 MPa for the water-soaked (24 h) and 0.55 MPa for the water-boiled (6 h) samples.

Untreated specimens exhibited significantly higher bond shear strength than treated specimens after 24 h of water soaking and 6 hours of water boiling. The average loss of shear strength after soaking and boiling treatments was about 46% and 51%, respectively, compared to the untreated specimens. There are two reasons for such a reduction in strength. Wood is rich in OH groups and can easily absorb water. After water absorption by the wood cells, below the saturation range of the fibers, the wood or EWP tends to swell, causing swelling stresses in the glue line and reducing the shear strength of the composite [28,32]. In addition, MOC is considered a rigid cement whose durability can be affected by temperature and humidity fluctuations, which in turn can degrade the physical and mechanical properties of MOC-based products. In MOC, prolonged contact with water leaches the magnesium chloride from the structure, leaving only hydrated brucite (Mg(OH)_2_) as the binder phase, which has reduced the strength of the binder [22,31,33]. Importantly, the differences between the shear strength values of the treated samples were not significant, indicating that the MOC binder has good resistance even to boiling water. This is an important result, showing the potential of MOC-bonded plywood also for exterior applications. Studies have shown that free/released MgCl_2_ in hot water can generate and precipitate Mg(OH)Cl, which effectively improves the hygroscopic properties of MOC [34]. In particular, the bio-based adhesives based on tannin, soy, chitosan, etc., developed for EWP production showed very low water resistance (see Table 5), especially to boiling water, making their final application a major challenge [1,2]. However, none of the plywood samples bonded with MOC immersed in water for 24 h and boiled in water for 6 hours experienced delamination, indicating the promising potential of MOC compared to bio-based adhesives.

**Figure 5:**
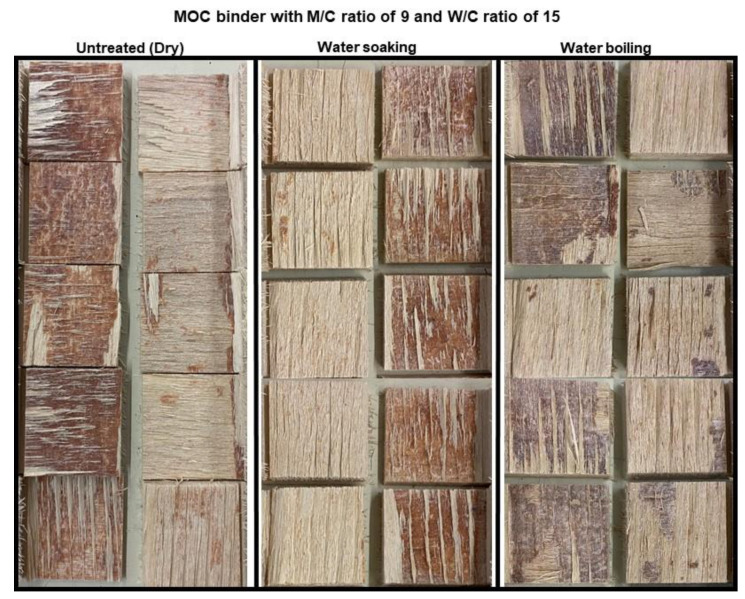
Fractured surface of the plywood bonded with MOC binder after shear strength test.

The shear strength values of the developed MOC-glued plywood must be compared with the values of EN 314-2 to determine the applicability of the boards. There is a correlation between the minimum shear strength value and the percentage of wood failure in EN 314-2. The higher the percentage of wood failure in the binder line, the lower the required value for shear strength. For example, if more than 40% of the binder line area fails, the required shear strength should be at least 0.6 MPa. In this study, the shear strength values of most specimens were below 1 MPa, which is the upper limit according to EN 314-2 if the wood does not fail at all. Treated specimens (immersed in tap water and boiled in water) with M/C and W/C ratios of 9 and 15, respectively, had a shear strength value of about 0.6 MPa and showed a maximum wood failure of about 30–35% (Figure 5). This is very close to the required values for interior and exterior applications according to EN314-2. In EWP, the performance and the type of the bonding mechanisms between the wood and the binder are considered to have a significant influence on the failure level of the wood after shear testing [28]. Since wood has a porous structure, mechanical interlocking is the most likely bonding mechanism between wood and minerals [11,12,13]. Chemical bonding between wood and minerals is only possible if organic–inorganic hybridization is used to enhance the binding mechanisms between minerals and wood, which needs further investigation [15,16,17,21,30].To fully meet the requirements of EN 314-2, the quality of the bond between the wood and the MOC binder can be improved by using organic binders, modifying the binder formulation and the parameters of the plywood manufacturing process.

#### 3.2.2. Bending Properties

**Figure 6:**
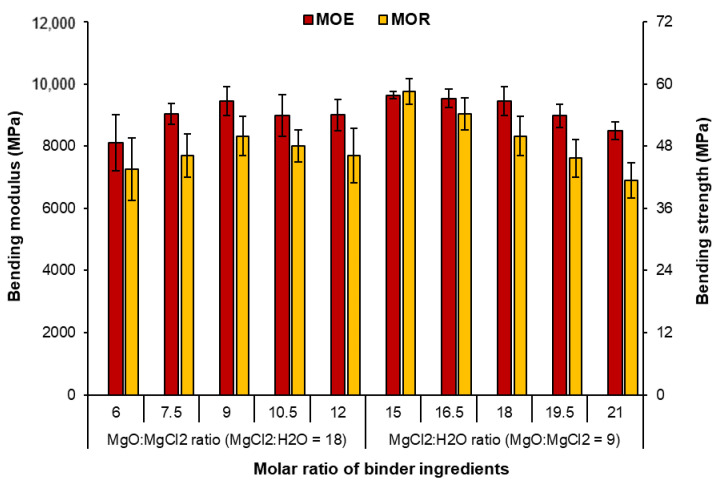
Bending properties of plywood samples using MOC binders with different molar ratios of the components.

Figure 6 shows that the molar ratio of M/C and W/C in the MOC binder affects the flexural properties of the plywood specimens. Plywood made from MOC binders with M/C and W/C molar ratios of 6 and 18, respectively, had MOE and MOR of about 8100 and 44 MPa, respectively. The MOE and MOR were increased by almost 15%, while the molar ratio M/C increased to 9. This may be related to the formation of more 5-phases, as in the hardened MOC binder with increased MgO content [22,23]. However, both MOE and MOR decreased when the M/C ratio reached a value of 12. A similar trend was also observed when the water content in the binder system was increased (increasing the molar ratio W/C). The higher the W/C molar ratio, the lower the binder strength and the lower the bending properties of the plywood panels. A more dilute binder can penetrate more into the wood structures, resulting in a starved binder line, which in turn has a negative effect on the bending properties [14,15,29]. Moreover, the further formation of Mg(OH)_2_ in the hardened binder leads to instability and a subsequent decrease in binder performance [24]. Excess and unconsumed water may have also caused pores and contributed to the observed reduction in strength [23]. 

The highest flexural strength and modulus of elasticity were observed for panels with M/C and W/C molar ratios of 9 and 15, respectively, with an MOE of about 9640 MPa and an MOR of about 59 MPa. The flexural properties of the MOC-bonded plywood are comparable to those of panels bonded with UF adhesive and other mineral and bio-based binders reported in the literature (the types of adhesives are listed in Table 5). In general, the MOC-bonded hot-press plywood exhibits comparable and even higher MOE and shear strength values than those reported in the literature. The slightly lower MOR values in this study can also be attributed to the lower density of the boards compared to the values reported in the literature.

**Table 5:** Selected properties of plywood bonded with different binder.

Wood Specie	Type of Binder	Standard Test Method ^2^	Flexural Modulus (MPa)	Flexural Strength (MPa)	Shear Strength (MPa)		Reference
					24 h Water Soaking	Water Boiling	
Poplar	UF	EN 314	9050	74	0.27	0	[13]
Beech	Tannin-furfural	EN 314	11,628	85	0	-	[35]
Poplar	Soy protein	GB/T 17657	-	-	0.35	-	[36]
Poplar	Chitosan	GB/T 17657	-	-	0.38	0.2	[37]
Poplar	Geopolymer	EN 314	8734	76	0.54	0.52	[38]
Poplar	MOC ^1^	GB/T 17657	-	-	0.88	-	[17]
Poplar	MOC	EN 314	9640	59	0.62	0.55	Current study

^1^ The binder contains 5% waterborne polyacrylamide, and the panels were produced by cold pressing. ^2^ Time for water boiling is 6 hours and 3 hours according to EN314 and GB/T 17657, respectively. (0) means that the specimens were delaminated before testing and (-) means that this test was not performed.

**Figure 7:**
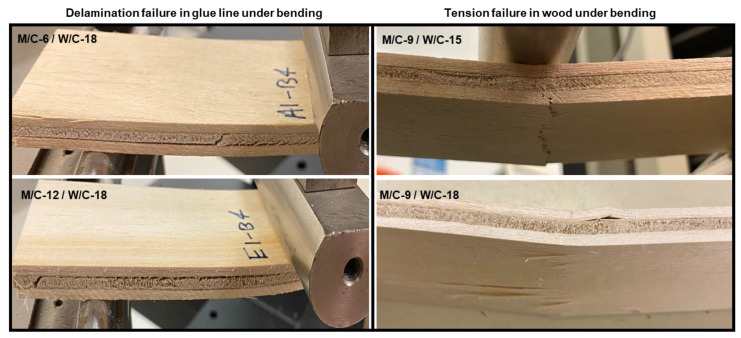
Failure modes of plywood samples during bending using MOC binders with different molar ratios of the components.

The failure modes of the flexural specimens are shown in Figure 7. The analysis of the failure modes can help to determine the relative impacts of the binder molar ratios on the bending properties of the plywood specimens. Shear failure in the binder line was the most frequently observed failure mode in flexural specimens. In other words, the binder line was delaminated and extended longitudinally along the entire length of the specimen [15]. This certainly influenced the flexural strength of the plywood specimens. In most plywood samples manufactured by MOC binders with a molar ratio of M/C of 9 and a molar ratio of W/C of 15 to 18, flexural failure (tensile failure in the wood) was induced in the wood layers, indicating improved adhesion in the binder line between the binder and the wood components. It is noteworthy that the flexural properties for these specimens were also the highest, which is consistent with the results of the shear strength test.

#### 3.2.3. Delamination Test

**Figure 8:**
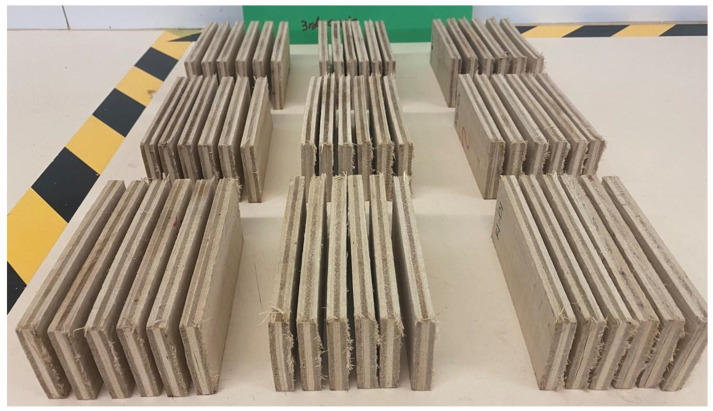
Plywood samples after third cycle of delamination testing according to ANSI/HPVA HP-1, 2020.

The delamination properties of the hot-pressed plywood with MOC as binder were also tested. Figure 8 shows pictures of samples after the delamination test. Remarkably, none of the samples delaminated, even after three soaking (4 h in tap water) and drying (19 h at 50 ± 1 °C) cycles. Considering the literature demonstrating that magnesium oxychloride cements exhibit low water resistance when exposed to water or humid conditions, these results are significant for further advances in MOC-bonded EWP. Although the exterior applications of these panels are acceptable according to these results and in accordance with ANSI/HPVA HP-1 (2020), the shear strength values (EN314-2) still need to be considered for such a conclusion.

### 3.3. Physical Properties

**Figure 9:**
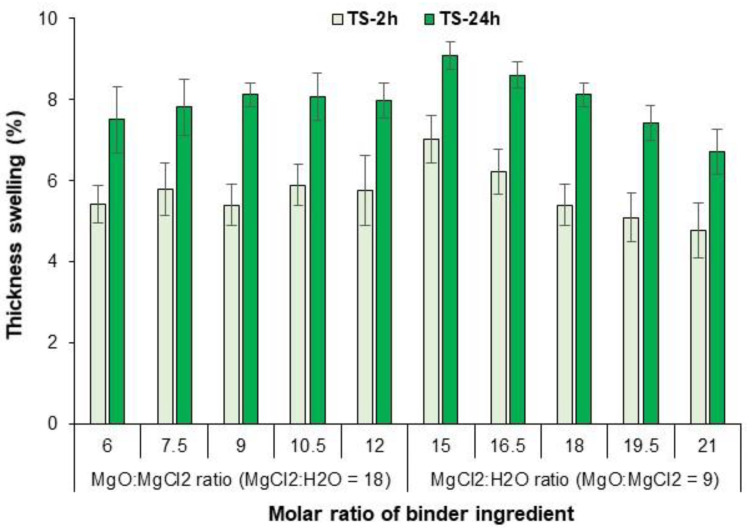
Thickness swelling of plywood samples during bending using MOC binders with different molar ratios of the components.

Figure 9 shows that increasing the molar ratio of M/C from 6 to 9 increases the TS, while further increasing M/C to 12 has no significant effect on TS values after both 2 and 24 h of water immersion. However, TS was significantly reduced by increasing the molar ratio of W/C from 15 to 21. Thickness swelling in wood and EWP is a complex property influenced by several factors such as wood species, density, adhesive formulation, the stress relaxation of the board and other manufacturing parameters and their interaction [26]. In this context, the accessibility of water molecules to the OH groups of the wood has the greatest influence on the final TS value of the EWP [7]. The accessibility of OH groups in the wood can be reduced by more penetration of the binder into the wood structure [13,14]. The solid content of the MOC binder is increased by increasing the M/C molar ratio and decreased by increasing the W/C molar ratio (Table 3). In other words, the lower the binder solid content, the higher the penetration of the binder into the wood cells and the lower the accessibility of the water molecules to the OH groups, so that the TS is reduced. In other words, a lower solids content of the binder leads to greater penetration of the binder into the wood structures and thus to less accessibility of the wood OH for water and to a reduction in the TS of the board. It is noteworthy that the TS after 24 h in all binder formulations is in the acceptable range and quite low, below 9%.

**Figure 10:**
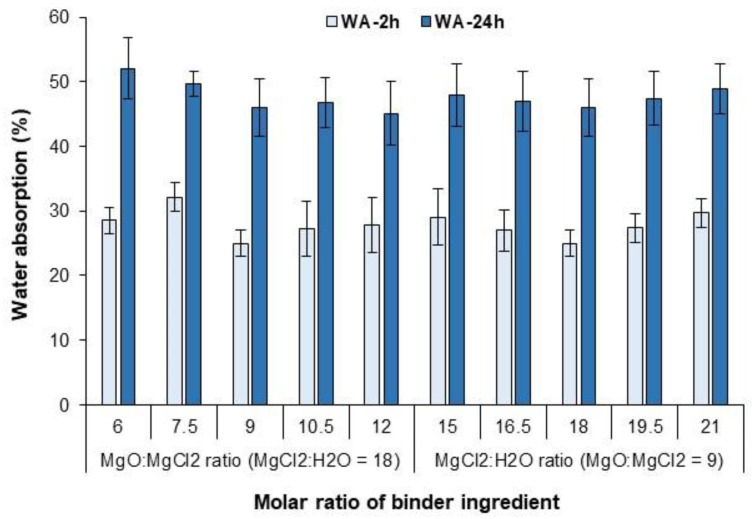
Water absorption of plywood samples during bending using MOC binders with different molar ratios of the components.

Figure 10 shows that the WA value did not change significantly at different molar ratios of M/C and W/C in the MOC binder formulation. For all binder formulations, the WA after 2 and 24 h of immersion in water was about 27% and 47%, respectively, which is similar to the conventional plywood panels bonded with urea-formaldehyde adhesive [13]. Apparently, all MOC binders had a dense microstructure at least during the 24 h water immersion and thus exhibited uniform moisture resistance. As also confirmed by the shear strength and delamination tests, the hot-pressed plywood with MOC as binder showed sufficient resistance to tap, and even hot water, where none of these specimens were delaminated. The curing of MOC at higher temperatures (e.g., 65 °C) resulted in a significant decrease in the moisture resistance of MOC, mainly due to the increased formation of 9-phases in MOC [23]. The 9-phases were found to be more susceptible to dissolution than the 5-phases, which formed predominantly at lower curing temperatures. It can be concluded that the duration of MOC hardening at elevated temperatures during hot pressing appears to have no or little impact on the moisture resistance of mineral-bonded plywood. Since hot pressing is the most expensive part of the entire production and is usually defined as a bottleneck, this finding is crucial for further progress in mineral-bonded plywood, especially with MOC as a binder.

## 4. Conclusions

A formaldehyde-free and non-fossil binder was developed to produce plywood, based on mineral components that are inherently resistant to fire and mildew. Considering the results of the current study, the production of plywood with MOC as a binder is possible using hot pressing technology. The characterization of the MOC binder showed that the MOC has a faster gel time than conventional UF adhesives and that its peak cure temperature is below 100 °C, which is desirable for hot press technology. Moreover, the M/C and W/C molar ratio of the MOC binder components showed an important influence on the curing phase, the reaction heat of the binder and, accordingly, on the shear strength of the plywood samples. Increasing the molar ratio M/C up to 9 had a positive effect on the performance of the plywood, but further increasing the M/C up to 9 and increasing the molar ratio W/C to 21 had a negative effect. Plywood panels produced with M/C and W/C molar ratios of 9 and 15, respectively, exhibited the best properties among all other binder formulations. In other words, M/C-9 and W/C-15 have a more stable binder phase and a denser microstructure. Importantly, the hot-pressed plywood with MOC as binder showed sufficient resistance to tap and even hot water, with none of the specimens (i.e., shear test, delamination test and water absorption) delaminated after exposure to water. It can be said that the boards can be suitable for both indoor and outdoor use, although their adhesion mechanisms with wood should be further improved by various techniques to achieve the level of shear strength and wood failure required by EN 314-2. To this end, the performance of the binder can be improved by using organic–inorganic hybridization and changing the parameters of the plywood manufacturing process.

## Figures and Tables

**Table 1:** Chemical compositions of the caustic burnt magnesium oxide.

Material	MgO	CaO	SiO_2_	Al_2_O_3_	Fe_2_O_3_
Magnesium oxide	92.60%	1.80%	1.33%	0.10%	0.48%

**Table 2:** Chemical compositions of the technical magnesium chloride.

Material	MgCl_2_	MgSO_4_	KCl	NaCl	CaCl_2_	Water
Magnesium chloride	47.0%	0.43%	0.67%	0.65%	0.05%	Approx. 51%

**Table 3:** Mixture composition of MOC binder.

Code *	Molar Ratio of MgO:MgCl_2_	Molar Ratio of H_2_O:MgCl_2_	Binder Solid Content (%)
M/C-6	6.0	18.0	50.8
M/C-7.5	7.5	18.0	55.0
M/C-9	9.0	18.0	58.5
M/C-10.5	10.5	18.0	61.5
M/C-12	12.0	18.0	64.1
W/C-15	9.0	15.0	62.9
W/C-16.5	9.0	16.5	60.6
W/C-18	9.0	18.0	58.5
W/C-19.5	9.0	19.5	56.6
W/C-21	9.0	21.0	54.7

* Code M/C-9 and W/C-18 were similar. M, C, W stand for MgO, MgCl_2_, H_2_O, respectively.

**Table 4:** Gel time, pH and differential scanning calorimetry (DSC) parameters of the MOC binders and UF as reference.

Code	pH	Setting Time (s)	DSC Parameters		
			Onset Temperature (°C)	Peak Temperature (°C)	Delta H (J/g)
M/C-6	8.65	155.3	82.7	98.3	342.8
M/C-9	8.57	139.3	81.7	97.4	370.9
M/C-12	8.62	116.3	80.8	96.7	375.6
W/C-15	8.29	124.7	84.8	99.3	269.9
W/C-18	8.57	139.3	81.7	97.4	370.9
W/C-21	8.96	142.0	82.3	97.9	461.6
UF *	8.05	213.3	77.5	92.8	126.0

* A total of 1% ammonium sulphate was added as hardener.

## Data Availability

The data presented in this study are available on request from the corresponding authors.
